# Identification and detection of pathogenic bacteria from patients with hospital-acquired pneumonia in southwestern Iran; evaluation of biofilm production and molecular typing of bacterial isolates

**DOI:** 10.1186/s12890-021-01773-3

**Published:** 2021-12-09

**Authors:** Farzad Mazloomirad, Sajad Hasanzadeh, Asghar Sharifi, Gordafarin Nikbakht, Narges Roustaei, Seyed Sajjad Khoramrooz

**Affiliations:** 1 Student Research Committee, Yasuj University of Medical Sciences, Yasuj, Iran; 2 Department of Internal Medicine, Yasuj University of Medical Sciences, Yasuj, Iran; 3 Cellular and Molecular Research Center, Yasuj University of Medical Sciences, Yasuj, Iran; 4 Department of Infectious Diseases, Yasuj University of Medical Sciences, Yasuj, Iran; 5 Department of Epidemiology and Biostatistics, School of Health and Nutrition Sciences, Social Determinants of Health Research Center, Yasuj University of Medical Sciences, Yasuj, Iran; 6 Department of Microbiology, Yasuj University of Medical Sciences, Yasuj, Iran

**Keywords:** Hospital-acquired pneumonia, *Acinetobacter baumannii*, Biofilms, Molecular typing, MRSA

## Abstract

**Background:**

Hospital-acquired pneumonia (HAP) is the second most common nosocomial infection in intensive care units (ICUs). The present study aims to determine the prevalence of pathogenic bacteria, their biofilm formation, and molecular typing from patients with HAP in southwestern Iran.

**Methods:**

Fifty-eight patients with HAP participated in this cross-sectional study. Sputum and endotracheal aspirate were collected from each patient for isolation and detection of bacteria. Biofilm formation was evaluated using Congo red agar or Microtiter plate assay. The antimicrobial susceptibility patterns of the isolates were investigated. The multiplex polymerase chain reaction (M-PCR) technique was used to determine the Staphylococcal Cassette Chromosome mec (SCC*mec*) types of methicillin-resistant *Staphylococcus aureus* (MRSA) strains. All *S. aureus* isolates were typed using the *agr* typing method. A repetitive element sequence-based PCR (rep-PCR) typing method was used for typing of Gram-negative bacteria. Data were analyzed using the Statistical Package for the Social Sciences (SPSS) software version 15 and the chi-square test.

**Results:**

Bacteria were isolated in 52 (89.7%) of patients. *Acinetobacter baumannii* (*A. baumannii*) was the most prevalent organism (37%), followed by *S. aureus*, *Pseudomonas aeruginosa* (*P. aeruginosa*), and *Escherichia coli* (*E. coli*). Using the PCR method, 56 bacteria were detected. *A. baumannii* was the most prevalent (35.7%) organism. *A. baumannii* and *P. aeruginosa* were biofilm-producing. All Gram-negative isolates were colistin-sensitive, and most of the *A. baumannii* isolates were multidrug-resistant (MDR). MRSA was identified in 12 (80%) *S. aureus* isolates, and 91.6% of MRSA were SCC*mec* type III. The agr type III was the most predominant. The rep-PCR analysis showed seven different patterns in 20 *A. baumannii*, six patterns in 13 *P. aeruginosa,* and four patterns in 6 *E. coli*.

**Conclusion:**

*A. baumannii* was more prevalent than *S. aureus* in ventilator-associated pneumonia (VAP), while *S. aureus* is a major pathogen in non-ventilator hospital-acquired pneumonia (NV-HAP), possibly due to the tendency of the former to aquatic environments. Based on the rep-PCR typing method, it was concluded that bacteria were transmitted from patients or healthcare workers among different wards. Colistin can be used as a treatment in Gram-negative MDR isolates.

## Background

Hospital-acquired pneumonia (HAP) is defined as a parenchymal lung infection not occurring at the time of hospitalization or during the incubation period but 48 h after hospital admission. HAP is the second most common nosocomial infection, leading to prolonged hospitalization, increasing costs, and high morbidity/mortality rates [1]. Common signs and symptoms include fever, leukocytosis, purulent secretions, increasing respiratory rates, abnormal chest examination, tachypnea, and impaired oxygenation [2]. HAP is divided into two subgroups: ventilator-associated pneumonia (VAP) and non-ventilator hospital-acquired pneumonia (NV-HAP) [3]. VAP, a subset of HAP, occurring 48 h or more after tracheal intubation and connected to the ventilator, is the most common infection in ICUs. NV-HAP occurs in patients hospitalized for at least 48 h and not connected to a ventilator [4, 5]. Bacterial infections are the leading cause of HAP. Among them, *Acinetobacter spp*, *P.aeruginosa*, *E. coli*, *Klebsiella pneumonia* (*K. pneumoniae*)*,* and *S. aureus* (especially MRSA) were the most common agents [6–8]. Among the major sources of these bacteria are hospital environments, patients’ microbial flora, and primary hospitalized patients [9].

Biofilm formation is one of the most effective factors in the pathogenicity of these bacteria. Biofilm may cause bacteria to develop antimicrobial resistance and interfere with host defense mechanisms. Biofilm can also be a reservoir for the recurrence of these pathogens [10]. An endotracheal tube is a potential reservoir for microorganisms to infect the respiratory tract, a risk factor for VAP, since microorganisms can adhere to its surface, and some species form biofilm thereon. These microorganisms may be transmitted to the lungs during suctioning [11]. Therefore, biofilm-producing bacteria can persist in hospital environments and significantly contribute to nosocomial infections (especially in HAP), leading to treatment failure, rising costs of treatment, and mortality in patients.

Molecular typing methods are widely used to identify infectious species for epidemiological studies, determine the population structure of microbial communities and genetic diversity within a species. Molecular typing of bacteria can effectively prevent and control infections. Repetitive element sequence-based polymerase chain reaction (rep-PCR) is a molecular typing method used to determine clonal relationships, genotyping, and phylogenetic relationships between closely related species [12, 13]. It is extensively used to identify, track, and study diversity in microorganisms [14]. Many typing methods are applied for *S. aureus* (an important nosocomial pathogen)*,* including multi locus sequence typing (MLST), *S. aureus* protein A (spa) typing, SCC*mec* typing, and accessory gene regulator (*agr*) typing. They can effectively describe epidemiologic trends and implement infection control strategies [15]. The *agr* locus is one of the major regulators of virulence factor production (hemolysins, enterotoxins, MSCRAMMs, etc.) in *S. aureus* [16]. Itis controlled by P2 and P3 promoters. The P2 operon consists of four genes: *agr*A, *agr*B, *agr*C, and *agr*D. The P3 operon transcribes a regulatory RNA molecule called RNAIII [17]. So far, four major *agr* groups (I–IV) have been identified based on the amino acid sequence polymorphism of the *agr*-encoded autoinducing peptide (*agr*D) and its corresponding receptor (*agr*C). The *agr* types are different in their properties and prevalence in various geographical areas. Thus, identifying predominant types in each region can serve epidemiological purposes. MRSA strains harbor the *mec*A gene and are located on the SCC. Based on complete sequence data, thirteen SCC*mec* types (I–XIII) have been defined in MRSA [18]. Among the various SCC*mec* types reported worldwide, types I-III are known as predominant HA-MRSA [19]. SCC*mec* typing can significantly contribute to the detection of strains associated with nosocomial infections [20].

Since microorganisms responsible for HAP differ between geographical regions and among hospitals or patients in one region and most empirically treated HAP, collecting information on bacterial pathogens and their antibiotic resistance patterns is necessary. Hence, the present study aimed to detect microorganisms from patients with HAP, evaluate biofilm production and antimicrobial susceptibility patterns of bacterial isolates, and conduct molecular typing (SCC*mec* typing, *agr* typing, and rep-PCR) of bacterial isolates collected from patients with HAP admitted in Shahid Beheshti and Imam Sajjad hospitals in southwestern Iran.

## Method

### Sampling method

This descriptive cross-sectional study was performed on 58 patients hospitalized in the Imam Sajjad and Shahi Beheshti hospitals affiliated to Yasuj University of Medical Sciences (YUMS) in southwest Iran from 2018 to 2019. The inclusion criteria are as follows: (1) should be above 15 years of age, (2) should have at least one or two of the following symptoms: fever, leukocytosis, leukopenia, purulent secretion of lungs, sputum, and cough, (3) should have been infected 48 h after hospitalization, and (4) for VAP, should have received mechanical ventilation 48 h or longer after intubation. Patients who were unwilling to enter into the study voluntarily, those suffering from immunodeficiency, and those showing signs of pneumonia within less than 48 h of hospitalization were excluded. The patients were diagnosed by a physician specializing in internal medicine and infectious diseases. The first morning sputum samples and endotracheal aspirate (ETA) were collected in sterile containers under aseptic conditions and delivered to the microbiology laboratory. The samples were then divided into two parts: the first part was inoculated on Mac Conkey agar (Condalab, Spain), blood agar (5% sheep), and chocolate agar (Condalab, Spain) media and incubated at 37 °C for 24–48 h. The bacteria were then identified based on Gram staining, colony morphology, and standard biochemical tests. The PCR method was applied for the final confirmation of isolated bacteria. The second part is stored at − 20 °C for molecular analyses. Demographic data, including age, sex, and clinical characteristics, were collected. Before sampling, informed consent was obtained from each patient. This study has been approved by the Research Ethics Committee of the Yasuj University of Medical Sciences (IR.YUMS.REC. 1396.194).

### Biofilm formation assay

For Gram-negative bacteria, biofilm formation was evaluated using the microtiter plate method [21]. First, each isolate was inoculated in Trypticase Soy Broth (TSB) and incubated at 37 °C for 24 h. Then, a 1:100 dilution of this suspension was prepared and 200 μl of this dilution was inoculated in sterile 96-well flat-bottom polystyrene microtiter plates. The negative control wells only contained sterile TSB medium and were incubated for 24 h at 37 °C (each test was repeated three times). Afterward, the contents of the wells were emptied, and each well was gently washed three times with 200 μl phosphate-buffered saline (PBS). The plates were then dried at room temperature, and 150 μl of 99% methanol were added to each well to fix the bacteria inside the wells. After 15 min, the contents of the wells were drained, and the plates were allowed to dry at laboratory temperature. The wells were then stained with 200 μl of 1% crystal violet for 20 min. They were then rinsed gently with water, to each 200 μl of acetic acid 33% was added to remove crystal violet. The plates were then incubated at 37 °C for 15 min, and the light absorption of the painted wells at 620 nm was read by enzyme-linked immunosorbent assay (ELISA). They were classified into four groups: strong biofilm producers, moderate biofilm producers, weak biofilm producers, and non-biofilm producers based on optical density (O.D.). For *S. aureus*, biofilm formation was evaluated using the Congo red method according to Avila-Novoa et al.’s method [22]. Black colonies yielded positive results. Weak biofilm-producing isolates remained pink, while occasional darkening at the centers of colonies was also observed. Bright red colonies were considered negative.

### Antimicrobial susceptibility testing

Antibiotic susceptibility pattern of isolates was performed using the disc diffusion method on Mueller Hinton Agar (Condalab, Spain) according to the Clinical and Laboratory Standards Institute (CLSI) guideline for the following antibiotics: penicillin (10 μg), cefepime (30 μg), cefoxitin (30 μg), sulfamethoxazole (23/75 μg), tazobactam (10 μg) + piperacillin (100 μg), cefotaxime (30 μg), ceftriaxone (30 μg), meropenem (10 µg), clindamycin (2 μg), clarithromycin (15 μg), levofloxacin (5 μg), azithromycin (15 μg) (BD-BBL Company, USA). The Minimum Inhibitory Concentration (MIC) of colistin and vancomycin was determined using the broth microdilution method. Bacterial isolates resistant to three or more different antimicrobial classes were identified as MDR isolates. *S. aureus* (ATCC 25923) and *E. coli* (ATCC 25922) were used as control strains.

### Molecular detection of bacterial pathogens

DNA was extracted from clinical specimens including sputum and ETA by utilizing the phenol–chloroform method and then stored at − 20 °C as DNA. This study employed the boiling method for bacterial isolates by the culture method. Table 1 lists the primers used in this study. The PCR program was as follows: the PCR protocol was carried out in a thermocycler (Bio-Rad, T100, USA) with an initial denaturation at 94 °C for 4 min, followed by 34 cycles of denaturation at 94 °C for 45 s, annealing at 55 °C for 45 s for *nuc*A, extension at 72 °C for 1 min, and a final cycle of extension at 72 °C for 5 min. For the detection of *A. baumannii*, *P. aeruginosa*, *E. coli,* and *K. pneumoniae,* the program was as follows: initial denaturation at 94 °C for 4 min, 30 cycles of denaturation at 94 °C for 45 s, annealing at 59 °C for 30 s for *glt*A and *opr*L*,* at 57 °C for 60 s for *aro*E and *ure*D, extension at 72 °C for 1 min, and a final cycle of extension at 72 °C for 5 min. The control strains included *S. aureus ATCC25923, K. pneumoniae ATCC 1290, E. coli ATCC 25922, P. aeruginosa PAO1, A. baummanii ATCC 19606*. The sequences of oligonucleotide primers for the detection of bacteria are shown in Table 1 [23–25].

**Table 1:** The primers oligonucleotide sequences used in this study

Target gene	Primer sequence (5′ → 3′)	Amplicon Length, bp	References
*nuc*A	F-CTG GCA TAT GTA TGG CAA TTG TT R-TAT TGA CCT GAA TCA GCG TTG TCT	670	[23]
*aro*E	F-AAGGTGCGAATGTGACGGTG R-AACTGGTTCTACGTCAGGCA	620	[24]
*ure*-D	F-CCC GTT TTA CCC GGA AGA AG R-GGA AAG AAG ATG GCA TCC TGC	243	[25]
*Opr*L	F-TTCCGGTGAAGGTGCCAAT R-ACCGGACGCTCTTTACCATA	507	Unpublished data
*glt*A	F-GACATCAACCACCGCGAAAT R-GGACCCCAAAGAGCAGAGAT	332	Unpublished data
*mec*A	F-GTG AAG ATA TAC CAA GTG ATT R-ATG CGC TAT AGA TTG AAA GGA T	147	[23]
SCC*mec* I	F-GCTTTAAAGAGTGTCGTTACAGG R-GTTCTCTCATAGTATGACGTCC	613	[26]
SCC*mec* II	F-CGTTGAAGATGATGAAGCG R-CGAAATCAATGGTTAATGGACC	398	[26]
SCC*mec* III	F-CCATATTGTGTACGATGCG R-CCTTAGTTGTCGTAACAGATCG	280	[26]
SCC*mec* IVa	F-GCCTTATTCGAAGAAACCG R-CTACTCTTCTGAAAAGCGTCG	776	[26]
SCC*mec* IVb	F-TCTGGAATTACTTCAGCTGC R-AAACAATATTGCTCTCCCTC	493	[26]
SCC*mec* IVc	F-ACAATATTTGTATTATCGGAGAGC R-TTGGTATGAGGTATTGCTGG	200	[26]
SCC*mec* IVd	F-CTCAAAATACGGACCCCAATACA R-TGCTCCAGTAATTGCTAAAG	881	[26]
SCC*mec* V	F-GAACATTGTTACTTAAATGAGCG R-TGAAAGTTGTACCCTTGACACC	325	[26]
*agr* I	R-GTCACAAGTACTATAAGCTGCGAT	440	[27]
*agr* II	R-GTATTACTAATTGAAAAGTGCCATAGC	573	[27]
*agr* III	R-CTGTTGAAAAAGTCAACTAAAAGCTC	406	[27]
*agr* IV	R-CGATAATGCCGTAATAC CCG	588	[27]
pan-agr	F-ATGCACATGGTGCACATGC		[27]

### Molecular typing (SCCmec typing and agr typing)

Multiplex PCR conditions were as follows: an initial denaturing at 94 °C for 4 min, followed by 35 cycles of denaturation at 94 °C for 45 s, annealing at 60 °C for 45 s for SCC*mec* types I, II, III, and V, and at 60.5 °C for 45 s for SCC*mec* subtypes IVa, IVb, IVc, and IVd, and extension at 72 °C for 40 s. For *agr* typing, the program was similar to *nuc*A except for annealing at 57 °C for 1 min. The final extensions were continued at 72 °C for 5 min and performed in a thermocycler (Bio-Rad, T100, and the USA). The following MRSA strains were used as positive controls: COL (SCCmec type I); XU642 (EMRSA-16, SCCmec type II); WBG525 (EMRSA-1, SCCmec type III3); and WBG9465 (EMRSA-15, SCCmec type IV2), kindly provided by Dr. Mohammad Emaneini. The sequences of oligonucleotide primers for the detection of SCC*mec* types and *agr* typing are shown in Table 1 [26, 27].

### Repetitive sequence-based polymerase chain reaction

The primers REP1 (5′-GCGCCGICATCAGGC-3′) and REP2 (5′- ACGTCTTATCAGGCCTAC-3′) were used for repetitive sequence-based polymerase chain reaction (rep-PCR). Amplification reactions were performed in a final volume of 25 μL, containing 12.5 µl Master Mix (Amplicon, Denmark), 25 ρmol of each primer, and 5 µl bacterial DNA. The PCR protocol included an initial denaturation at 94 °C for 10 min, followed by 30 cycles of denaturation at 94 °C for 1 min, annealing at 45 °C for 1 min, extension at 72 °C for 1 min, and a final extension at 72 °C for 16 min. The PCR products were electrophoresed on a 2% agarose gel at 90 V for 45 min (for rep-PCR 60 V for 150 min), stained with DNA Safe Stain, and visualized under UV light using a gel documentation system (Major Sciences, Taiwan).

## Results

### Demographic and clinical characteristics of patients

A total of 58 patients participated in the study. At least one bacterial isolate was identified in 52 (89.7%) patients using the culture method. The average age of culture-positive patients was 57.92 ± 18.59 years (ranging between 18 and 94), 52% of whom were female. Besides, 36 (69%) patients were VAP, and 16 (31%) were NV-HAP. The most common clinical symptoms and laboratory results were leukocytosis (100%), fever (82.7%), and sputum (82.7%). Table 2 presents the demographic and clinical characteristics of patients with VAP and NV-HAP.

**Table 2:** Demographic and clinical characteristics of patients with HAP

	Culture positive			Culture negative
	Total (N = 52)	VAP (N = 36)	NV HAP (N = 16)	Total (N = 6)
*Sex and age*				
Female	27 (51.9%)	17 (47.2%)	10 (62.5%)	2 (33.3%)
Male	25 (48.1%)	19 (52.8%)	6 (37.5%)	4 (66.7%)
Average age (YRS)	57.92	59.39	54.64	62.5
*Clinical presentation*				
Fever	43 (82.7%)	30 (83.3%)	13 (81.2%)	5 (83.3%)
Sputum	43 (82.7%)	30 (83.3%)	13 (81.2%)	4 (66.7%)
Chest pain	15 (28.8%)	9 (25%)	6 (37.5%)	2 (33.3%)
Cough	10 (19.2%)	7 (19.4%)	3 (18.7%)	2 (33.3%)
Underlying disease	20 (38.4%)	14 (38.9%)	6 (37.5%)	1 (16.7%)

### Detection of bacteria using the culture and PCR methods

Using the culture method, 54 bacterial isolates were collected from 52 patients. Among them, Gram-negative bacilli were obtained in 39 (72.2%) and Gram-positive cocci (*S. aureus*) in 15 (27.8%). Among Gram-negative bacteria, *A. baumannii* was obtained in 20 (37%), *P. aeruginosa* in 13 (24.1%), and *E. coli* in 6 (11.1%) patients. Most bacterial isolates from patients with VAP were Gram-negative (84.6%), while 56.3% were prominent in NV-HAP Gram-positive bacteria (*S. aureus*). *A. baumannii* was the most predominant isolate identified in 37% of patients. *A. baumannii* was identified more in patients with VAP than those with NV-HAP, and the difference was statistically significant (*P* = 0.016). Moreover, *S. aureus* was detected more in patients with NV-HAP than those with VAP, and the difference was statistically significant (*P* = 0.003). (More details are shown in Table 3).

**Table 3:** Bacterial identified in this study from patients with VAP and NV-HAP using culture and PCR methods

Bacteria	VAP		NV-HAP		*P* value*	*P* value**
	Culture (N = 38)	PCR (N = 39)	Culture (N = 16)	PCR (N = 17)		
*Acinetobacter baumannii*	18 (47.4%)	18 (46.2%)	2 (12.5%)	2 (11.8%)	0.016	0.014
*Pseudomonas aeruginosa*	10 (26.3%)	10 (25.6%)	3 (18.7%)	3 (17.6%)	0.554	0.518
*Staphylococcus aureus*	6 (15.8%)	6 (15.4%)	9 (56.3%)	9 (52.9%)	0.003	0.004
*Escherichia coli*	4 (10.5%)	5 (12.8%)	2 (12.5%)	2 (11.8%)	0.832	0.918
*Klebsiella pneumonia*	0	0	0	1 (5.9%)	–	–
Total	100%	100%	100%	100%		

^*^Between the cultures of the two groups VAP and NV-HAP

^**^Between the PCR of the two groups VAP and NV-HAP

Using the PCR method, 56 bacteria were detected, 69.6% from patients with VAP and 30.4% from those with NV-HAP. The detection rate of microorganisms was as follow: 35.7% (N = 20) for *A. baumannii*, 26.8% (N = 15) for S*. aureus*, 23.2% (N = 13) for *P. aeruginosa*, 12.5% (N = 7) for *E. coli*, and 1.8% (N = 1) for *K. pneumoniae.* There were no significant differences between bacteria isolated by either the culture or PCR methods.

### Biofilm production

Totally, 94.4% of the isolates were biofilm-producing. Among them, the highest rate of biofilm formation was observed in *A. baumannii* and *P. aeruginosa* (100%), followed by *S. aureus* (86.7%) and *E. coli* (83.4%) (Table 4).

**Table 4:** Biofilm production of bacterial isolates in the present study from patients with HAP

	Microtiter plate method			Congo red agar method	Total (N = 54)
	*Acinetobacter baumannii* (N = 20)	*Pseudomonas aeruginosa* (N = 13)	*Escherichia coli* (N = 6)	*Staphylococcus aureus* (N = 15)	
Strong biofilm producer	18 (90%)	12 (92.3%)	3 (50%)	7 (46.7%)	40 (74%)
Moderate biofilm producer	2 (10%)	1 (7.7%)	1 (16.7%)	4 (26.7%)	8 (14.8%)
Weak biofilm producer	–	–	1 (16.7%)	2 (13.3%)	3 (5.6%)
Negative biofilm	–	–	1 (16.6%)	2 (13.3%)	3 (5.6%)

### Antibiogram

Among Gram-negative bacteria, the highest rate of resistance was observed in levofloxacin, cefotaxime, and ceftriaxone. *S. aureus* isolates have a high rate of resistance to levofloxacin and penicillin. Table 5 presents the antibiotic resistance patterns of the Gram-positive and Gram-negative bacterial isolates. All of the Gram-negative isolates were susceptible to colistin.

**Table 5:** The antibiotic resistance patterns of the Gram-positive and Gram-negative bacterial isolates from patients with HAP

Antibiotics	*Staphylococcus aureus*	*Acinetobacter baumannii*	*Escherichia coli*	*Pseudomonas aeruginosa*
Penicillin	93%	–	–	–
Cefoxitin	80%	–	–	–
Clarithromycin	73%	–	–	–
Sulfamethoxazole	60%	–	–	–
Vancomycin	0%	–	–	–
Clindamycin	73%	–	–	–
Azithromycin	80%	–	–	–
Levofloxacin	80%	100%	83.3%	71.4%
Colistin	–	0%	0%	0%
Cefepime	–	38.1%	66.7%	35.7%
Ceftriaxone	–	100%	66.7%	78.6%
Meropenem	–	95.2%	33.3%	64.3%
Cefotaxime	–	100%	66.7%	85.7%
Piperacillin + Tazobactam		100%	33.3%	64.3%

### SCCmec typing

The *mec*A gene was identified in 12 (80%) *S. aureus* isolates and defined as MRSA, among which 11 cases (91.6%) harbored SCC*mec* type III (the most frequent SCC*mec* type) and 1 case (8.4%) harbored SCC*mec* type I. Types II, IVa, IVb, IVc, IVd, and V were not recognized in any isolates.

### Agr typing

Among 15 *S. aureus* isolates, 73% (N = 11) were successfully typed using the *agr* typing method. *agr* type III was the most prevalent *agr* type identified in 54% (N = 6), followed by *agr* type I (36%, N = 4) and *agr* type II (9%, N = 1). No *agr* types IV were observed in the isolates.

### Rep-PCR typing

**Fig. 1:**
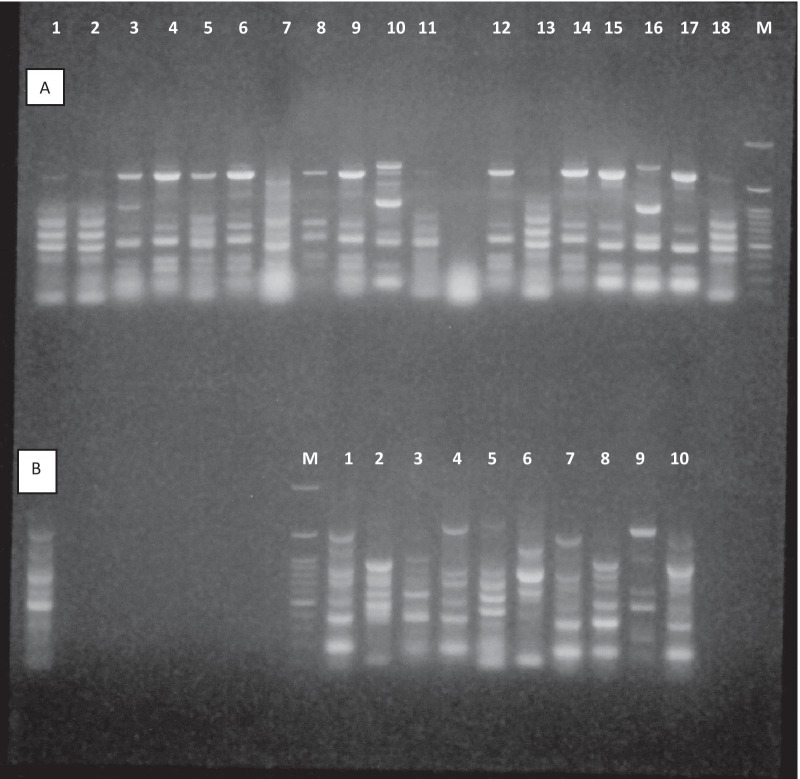
Molecular typing of Gram-negative isolates using rep-PCR method. **A** Showed different patterns in *A. baumannii*, **B** showed different patterns in *P. aeruginosa*

In Gram-negative bacteria, the rep-PCR typing pattern of isolates (based on band pattern similarity) generally exhibited great genetic diversity among isolates. The rep-PCR analysis showed seven different patterns in 20 *A. baumannii* isolates, six patterns in 13 *P. aeruginosa* isolates*,* and four patterns in 6 *E. coli* isolates (Table 6 and Fig. 1).

**Table 6:** Typing of Gram-negative bacteria based on rep- PCR typing method from patients with HAP

Bacterial isolated	A	B	C	D	E	F	G
*Acinetobacter baumannii*	1, 2, 124, 14, 19	4, 6, 8, 9, 13, 15	3, 10, 17	5, 11	16, 18	7	12
*Pseudomonas aeruginosa*	2, 5, 11	3, 6	4, 9	7	8	10	–
*Escherichia coli*	1, 2	3, 5	4	7	–	–	–

## Discussion

HAP is a common, serious, and costly problem in hospitals with dangerous complications and an important cause of death in patients admitted to the ICUs [28–30]. In the United States, HAP occurs at a rate of 5–20 cases per 1000 hospital admissions [31]. In various studies, Gram-negative bacteria are more prevalent than Gram-positive bacteria in HAP [32–34]. In the present study, Gram-negative bacteria (*A. baumannii*, *P. aeruginosa*, *E. coli,* and *K. pneumoniae*) were isolated at a rate of 72.2%. The frequency of HAP-causing bacteria may vary by hospital, geographic area, patient status, immune system, and health status. *A. baumannii* is an opportunistic pathogen associated with nosocomial infections, especially in ICUs. In the present study, like previous studies [35–38], *A. baumannii* was the most common microorganism isolated from HAP, implying the important role of *A. baumannii* in HAP. The high percentage of *A. baumannii* isolates in this study indicated its colonization capacity (in hospital environments, staff hands, and patient equipment), biofilm formation, the transmission of organisms to lower airways during suctioning in ventilated patients. *S. aureus* plays an important role in nosocomial infections, especially in HAP. Herein, it was the second organism(27.8%), similar to Ronald et al. [39] study, whose reported values were higher than in other studies [32, 40, 41]. The presence of *S. aureus* in hospital environments and its long-term colonization capacity may be attributed to microbial surface components recognizing adhesive matrix molecules (MSCRAMMs), biofilm formation, and teichoic acid (TA). In accordance with our study, Feng et al. detected *A. baumannii* in patients with VAP rather than those with NV-HAP, while *S. aureus* was detected in patients with NV-HAP more than those with VAP [8]. This can be due to the fact that *A. baumannii* tends to aquatic environments. *P. aeruginosa*, one of the most common opportunistic pathogens leading to severe nosocomial infections and a stable pathogen that easily forms biofilm and colonizes the body, was the third (24%) isolated pathogen in the present study. *A. baumannii* and *P. aeruginosa* were the most common isolated organisms in patients with VAP, which can be due to their tendency to aquatic environments. Herein, *E. coli* was isolated from11.2% of patients with HAP*,* higher than reported earlier [37, 39, 41]. In contrast, *K. pneumoniae* was not isolated in any case using the culture method, unlike many earlier studies [37, 40, 42].

The development of nosocomial infections in hospitalized patients (especially in the ICUs) is usually associated with ventilators, venous catheters, and urinary catheters. Many infections caused by these devices are related to contamination caused by biofilm-producing microorganisms. In general, colonization and biofilm formation are two important factors in HAP-causing bacteria [10]. Herein, 94% of the isolates were able to form biofilm. Additionally, all of the *A. baumannii* and *P. aeruginosa* isolates were biofilm-producing, similar to the studies by Asadian et al. [43] and Alonso et al. [44], indicating the significant role of biofilm in HAP-causing bacteria. Totally,86.7% of the *S. aureus* isolates were biofilm-positive, higher than what was reported by El-Nagdy [45]. Endotracheal tube, a potential source of intubated patients’ pulmonary contamination. It can cause damage to and transfer bacteria from the pharyngeal cavity to the lower airways, on which biofilm can form by bacteria, which is then removed during suctioning and delivered to the lower device [11]. In addition, microorganisms can be established due to biofilm and colonization in hospital environments for longer time periods. Biofilm formation in bacteria can lead to increased antibiotic resistance, development of chronic and persistent infection, and increased mortality rates in patients with HAP. Therefore, preventing biofilm formation requires the surveillance of hygiene standards, appropriate replacement of endotracheal tube, and proper sterilization of medical instruments.

The rep-PCR analysis showed seven different patterns in 20 *A. baumannii* isolates, six patterns in 13 *P. aeruginosa* isolates*,* and four patterns in 6 *E. coli* isolates, suggesting high pattern diversity among bacterial isolates, which could be related to the transmission of bacteria from patients or healthcare workers among different wards. Analyzing the rep-PCR typing method in Gram-negative bacteria demonstrated that bacterial isolates with similar patterns are isolated from patients in consecutive intervals caused by a common source of infection.

Herein, 80% of *S. aureus* harbored the *mec*A gene and was identified as MRSA, which is in accordance with the studies by Abbasi-Montazeri et al. [46] and Khoshnood et al. [47], which was lower than what was reported by Mohammadi et al. [48]. Like earlier studies, SCC*mec* type III was the most prevalent [49, 50]. However, in a study by Abbasi Montazeri et al., SCC*mec* type I was identified as predominant in their methicillin-resistant coagulase-negative staphylococci (MR-CoNS)) isolates [51]. The predominance of SCC*mec* type III suggested that the *S. aureus* isolates in the present study are of hospital origin. Hence, the Infection Control and Prevention Program (ICPP) is necessary to limit or eradicate the bacteria circulating in hospitals.

The majority of the *S. aureus* isolates belonged to *agr* type III. In accordance with our study, two recent studies [52, 53] reported that *agr* group III was the most predominant in their isolates, while in other studies, *agr* type I and II [54] and *agr* type I and III [55] were the most common. Our findings revealed that the *S. aureus* isolates with *agr* group III were more prevalent in nosocomial infections.

Antibiotic resistance is turning into a public health crisis, especially in developing countries. The highest rate of resistance was observed among Gram-negative bacteria to levofloxacin, cefotaxime, and ceftriaxone. Most of the *A. baumannii* isolates in this study were MDR*.* The highest rate of antibiotic resistance was observed to levofloxacin, ceftriaxone, meropenem, cefotaxime, and piperacillin-tazobactam, which is consistent with other studies indicating that most of the *A. baumannii* isolates were MDR [42, 56, 57].

More than 60% of *P. aeruginosa* isolates were resistant to levofloxacin, ceftriaxone, meropenem, cefotaxime, piperacillin-tazobactam*.* Unlike this study, Delle Rose et al. [58] from Italy reported that only 33.3% of *P. aeruginosa* isolates were resistant to meropenem. They also concluded that 31.2%, 25%, and 45% of the isolates were resistant to levofloxacin, ceftriaxone, and tazobactam, respectively, which is lower than that reported in our study. In comparison, in another study from India by Tiwari et al. [42], 80% of their isolates showed resistance to meropenem. In general, the high antibiotic resistance rate of *P. aeruginosa* and *A. baumannii* makes treatment difficult. According to the studies, the proper antibiotic selection is challenging because these bacteria are resistant to different antibiotics by using various antibiotic resistance mechanisms. All Gram-negative bacteria herein were colistin-sensitive, similar to studies by Moosavian et al. [59] and Sharifi et al. [60]. It can be used as a treatment against MDR Gram-negative isolates. Varying antibiotic resistances in different studies may be related to healthcare policies in different countries, length of hospital stay, and the geographical distribution of bacterial isolates. The present study results indicated a dramatic increase in the rate of antibiotic resistance, which could be due to antibiotic overuse, empiric therapy without antibiogram results, lack of proper sterilization of ventilators after each use, and use of non-standard ventilation systems.

## Conclusions

The most common isolated bacteria in HAP were *A. baumannii*, *S. aureus*, *P. aeruginosa,* and *E. coli*. Among them, *A. baumannii* was more prevalent in patients with VAP. Meanwhile, *S. aureus* is a major pathogen in patients with NV-HAP, which could be related to the tendency of *A. baumannii* to aquatic environments. Most bacterial isolates were biofilm-producing, leading to increased antibiotic resistance and chronic and persistent infection in HAP. All Gram-negative bacteria isolated herein were sensitive to colistin, which can be used to treat MDR Gram-negative isolates. Using the rep-PCR typing method, high diversity of patterns were observed among bacterial isolates, which may be due to the transmission of bacteria from patients or healthcare workers among different wards.

## Data Availability

The datasets used and/or analyzed during the current study are available from the corresponding author on reasonable request.
